# Cancer Chemoprevention Using Nanotechnology-Based Approaches

**DOI:** 10.3389/fphar.2020.00323

**Published:** 2020-04-03

**Authors:** Preshita Desai, Naga Jyothi Thumma, Pushkaraj Rajendra Wagh, Shuyu Zhan, David Ann, Jeffrey Wang, Sunil Prabhu

**Affiliations:** ^1^Department of Pharmaceutical Sciences, College of Pharmacy, Western University of Health Sciences, Pomona, CA, United States; ^2^Department of Pharmaceutics, College of Medicine, Jiaxing University, Jiaxing, China; ^3^Department of Diabetes and Metabolic Diseases Research, Beckman Research Institute, City of Hope, Duarte, CA, United States

**Keywords:** chemoprevention, nanotechnology, targeted delivery, efficacy, relapse

## Abstract

Cancer research in pursuit of better diagnostic and treatment modalities has seen great advances in recent years. However, the incidence rate of cancer is still very high. Almost 40% of women and men are diagnosed with cancer during their lifetime. Such high incidence has not only resulted in high mortality but also severely compromised patient lifestyles, and added a great socioeconomic burden. In view of this, chemoprevention has gained wide attention as a method to reduce cancer incidence and its relapse after treatment. Among various stems of chemoprevention research, nanotechnology-based chemoprevention approaches have established their potential to offer better efficacy and safety. This review summarizes recent advances in nanotechnology-based chemoprevention strategies for various cancers with emphasis on lung and bronchial cancer, colorectal, pancreatic, and breast cancer and highlights the unmet needs in this developing field towards successful clinical translation.

## Introduction

Cancer poses a severe socioeconomic impact all over the world. As per the American Cancer Society, over 1,762,450 new cancer cases and about 606,880 cancer related deaths are estimated in the United States alone in 2019 (average of 4,830 new cases and 1,660 deaths per day) (American Cancer Society, 2019a). Late/limited diagnostic opportunities, faster progression, limited treatment options with severe toxicities, and frequent relapse are the key reasons for high cancer fatality and is an alarming concern despite extensive research conducted in this field and available clinical treatments thus far. Therefore, alternatives like cancer chemoprevention, which focus on reducing cancer incidence and/or relapse, are gaining wide attention in recent years. Cancer chemoprevention can be looked upon as a strategy designed to minimize and delay the incidence, progression, or relapse of cancer. The objective is to interfere with the process of carcinogenesis so as to arrest or substantially retard the growth of precancerous lesions to reduce cancer incidence (Patterson et al., 2013).

Over the decades, literature reports few hundreds of molecules to elicit such chemopreventive potential and among them the widely reported drug classes are nonsteroidal anti-inflammatory drugs or NSAIDS (e.g., aspirin, ibuprofen, etc.); antioxidants [curcumin, ferulic acid, resveratrol, ellagic acid, epigallocatechin-3-gallate (EGCG), etc.]; extracts from natural origin (tea, wheat bran, etc.); minerals and ions (calcium, zinc, etc.). However, very few have been clinically approved under the broader umbrella of chemoprevention strategies with a listed use to treat or mitigate risk of precancerous lesions (Patterson et al., 2013). Evidently, the clinical success of these actives is restricted due to drug delivery challenges. Hence, use of smart nano drug delivery systems ensuring *in vivo* absorption and transportation of such actives at chemoprevention sites using passive and active targeting approaches is gradually coming on the forefront (Siddiqui et al., 2012; Desai et al., 2019a; Desai et al., 2019b). Though nanochemoprevention research is in its infancy, its potential is evident from increasing research in the field and reported scientific literature. This review summarizes recent advances in this niche field and highlights the unmet needs towards successful clinical translation.

### Role of Nanotechnology in Chemoprevention

**Figure 1:**
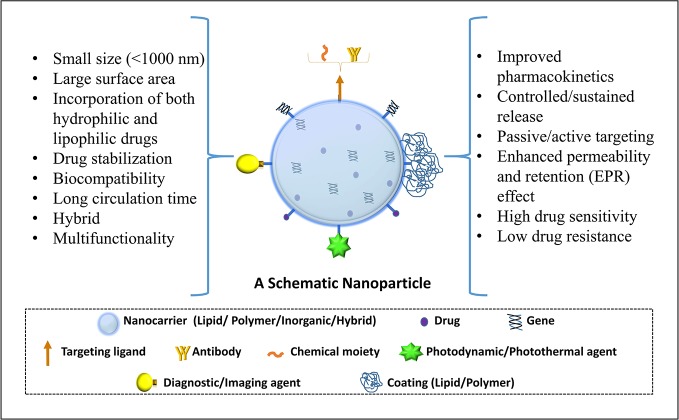
Schematic representation of a functional nanocarrier and its superior properties.

**Figure 2:**
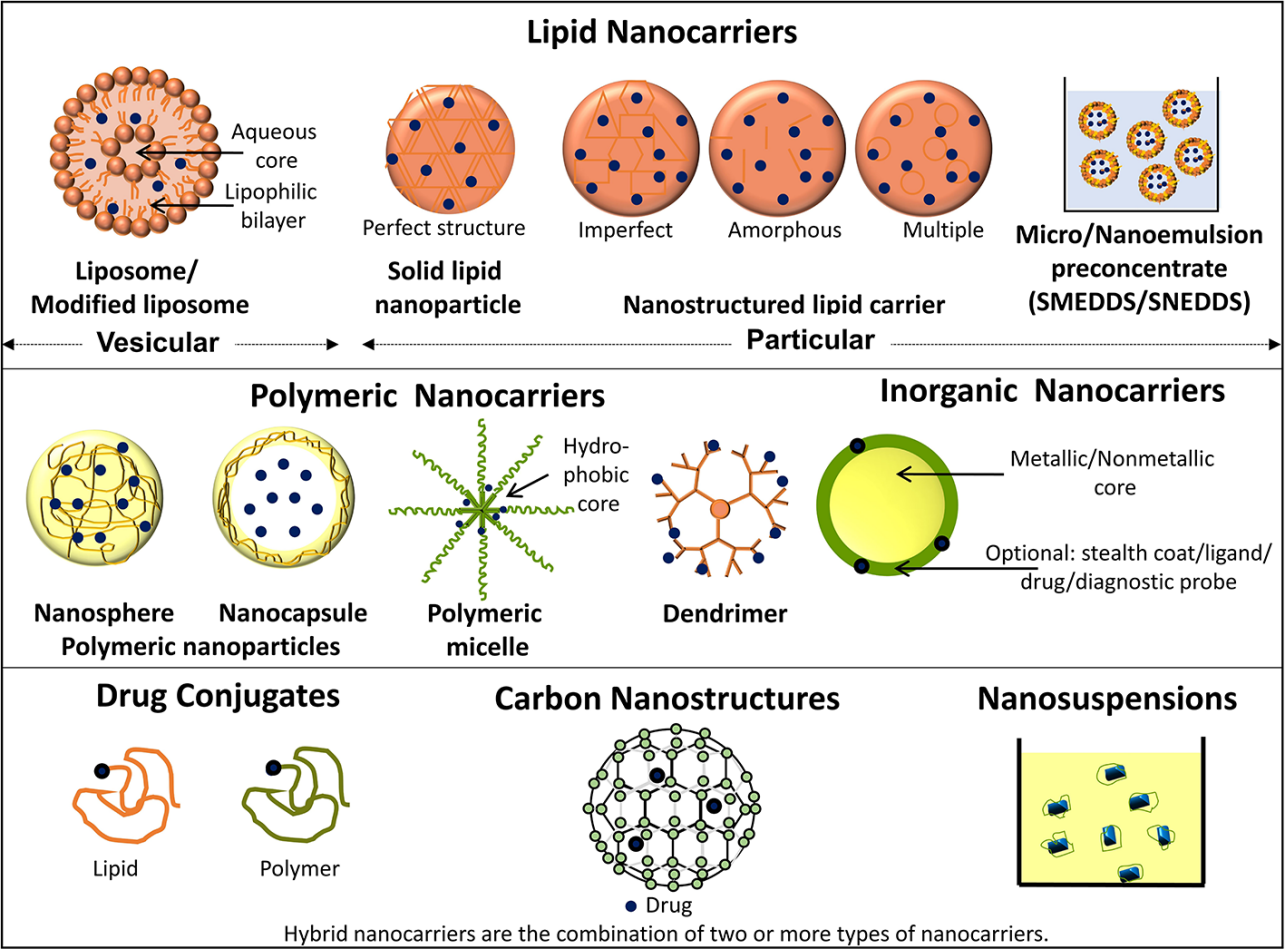
Schematic representation depicting types of nanocarriers.

Nanotechnology-based products broadly refer to nanoformulation comprising of particles ≤100 nm and from literature perspective those <1000 nm (Jeevanandam et al., 2018). Such nanoscale size elicits superior properties to these drug carrier system from absorption, targeting, and safety aspect which are summarized in **Figure 1** (modified from Desai et al., 2019a). Briefly, the overall drug efficacy and safety depend upon its intrinsic potency and variable factors like drug pharmacokinetics, toxicity, targeted delivery, and stability. Nanotechnology-based drug carriers successfully enhance these variable properties and thereby increase drug efficacy. Further, implementation of nanotechnology-based formulation in cancer prevention and therapy becomes very important in view of chemotherapy-associated side effects as they can provide an opportunity for possible dose reduction and drug targeting which can additionally enhance drug safety by minimizing off-target toxicities. Nanoformulations can be broadly classified based on their excipient composition and are depicted in **Figure 2** (modified from Desai et al., 2019a), which can be polymeric, lipid, carbon based, inorganic, or combinations thereof (Muqbil et al., 2011; Siddiqui et al., 2012; Miller et al., 2016).

### Nanotechnology-Based Chemoprevention Approaches

#### Lung and Bronchial Cancer

Lung and bronchial cancer is the leading cause of cancer-related deaths in the United States (American Cancer Society, 2019a). The key carcinogenesis factor is long-term tobacco use while other factors include exposure to radon gas, asbestos, air pollution, and second-hand smoke (Office on and Health, 2006; Gemine et al., 2019). The long-term prevention strategy is smoking withdrawal but the cancer risk of prior or current smoking population remains high. The average age of cohort diagnosed with lung cancer is 50–75 years with majority of patients being 65 years or older. The existing treatment modalities (surgery, chemotherapy, or radiation therapy) have succeeded in elongating life expectancy but in most cases, the disease is incurable leading to high fatality (American Cancer Society, 2019b; Farr et al., 2019; Mieras et al., 2019). Chemoprevention has been explored for management of lung cancer using natural or synthetic compounds to inhibit progression or suppress, reverse tumor growth. To overcome hydrophobicity and low bioavailability of such actives, nanotechnology-based approaches have been investigated. One such hydrophobic active is luteolin from green vegetables. Majumdar et al. developed nanoluteolin comprising luteolin nanocapsules with water-soluble polymer. They reported enhanced chemoprevention efficacy with nanoluteolin in an *in vitro* setting using cell lines of lung cancer (H292) and squamous carcinoma head and neck cancer (Tu212) and similar significant efficacy was observed in a tumor xenograft model (Emory Health Sciences, 2014; Majumdar et al., 2014). In another study, to enhance the solubility of chemopreventive antioxidant resveratrol, hydroxypropyl-β-cyclodextrin complex was developed for intranasal delivery. A 25-day *in vivo* study in A/J mice lung carcinogenesis model demonstrated 27% reduction in tumor multiplicity with 45% lower tumor volume confirming the efficacy of the developed formulation. Such a formulation approach is anticipated to enhance the drug bioavailability and hence has great potential in future clinical studies (Monteillier et al., 2018). In another study, lipid nanoparticles (NPs) of three chemopreventive drugs N-acetyl-L-cysteine, phenethyl isothiocyanate, and resveratrol were developed and their chemopreventive potential was assessed in bronchial epithelial cells. The study revealed significant enhancement in reducing the DNA fragmentation due to cigarette smoke with resveratrol lipid NPs confirming its potential to increase efficacy of lipophilic drug. However, the results were not significant with hydrophilic drug N-acetyl-L-cysteine indicating that appropriate selection of drug and NPs combination is very essential in development of successful chemoprevention strategy (Pulliero et al., 2015). Similarly, to enhance the *in vivo* performance of well-reported natural chemopreventive agent naringenin, polycaprolactone NPs of naringenin were developed with hyaluronic acid as an active targeting agent. The developed NPs were proven to show enhanced inhibitory potential against lung cancer cell line A549 but was found to be safe against J774 macrophage cell line confirming both enhanced efficacy and safety. The *in vivo* studies using urethane-induced lung cancer in rat model established significant tumor inhibitory activity confirming the *in vivo* drug targeting (Parashar et al., 2018). Further, a natural antioxidant, curcumin has shown a strong lung cancer stem cells suppression potency but its poor bioavailability makes it ineffective *in vivo*. Therefore, enhancing the bioavailability of drug is a potential approach and various NPs like lipid, polymer, liposomes have been reported to do so. Other promising agents such as vitamin A (retinoids or carotenoids), isothiocyanates, green tea extract, and bitter melon extract have also shown promise in head and neck cancer and their efficacy could be improvised to clinical significance using nanotechnology-based approaches.

#### Colorectal Cancer

Colorectal cancer is the second common cause of cancer related deaths in the United States (Americancancersociety, 2019a). Most of the colon cancers develop from the noncancerous adenomatous polyps, but when left untreated these polyps can become cancerous (Testa et al., 2018). Though surgical resection is the primary line of treatment, uncertainty in the detection methods and poor compliance leads to development of metastatic cancer and relapse (Vernon, 1997). Hence, prevention of polyp formation and development can be considered as a first line prevention approach.

For colon cancer chemoprevention, NSAIDs are most widely used (U.S. Preventive Services Task Force, 2007; Alizadeh et al., 2012; Drew et al., 2016; Pan et al., 2018). The epidemiological studies show that among all the NSAIDs, aspirin is the most promising agent in reducing adenomatous cancer recurrence due to the availability of remarkably consistent data with no cardiovascular risk and minimal gastrointestinal toxicity (Umezawa et al., 2019). Further, aspirin has also received a Grade B recommendation by the (U.S. Preventive Services Task Force, 2016) for its use as a chronic prophylaxis agent for colorectal cancer (Dehmer et al., 2015). Although it has been proved that aspirin alone or in combination have colon chemopreventive activity, nanoencapsulation of aspirin can further potentiate the efficacy with decreased dose. Chaudhary et al. studied chemopreventive effect of a mixture of aspirin, folic acid, and calcium on azoxymethane treated 7-week-old male Sprague-Dawley rats. The polymeric nanocapsules of drug combination prepared using polylactic-co-glycolic acid (PLGA) 50:50 polymer showed 1.7-fold more effective in chemoprevention than their unmodified counterpart regimen (Prabhu et al., 2007; Chaudhary et al., 2011). Celecoxib is another NSAID which is being widely explored in clinical setting for its chemoprevention potential but offers cardiotoxicity and pharmacokinetic variability (Solomon et al., 2005). Studies show that polymeric NPs of celecoxib prepared using ethyl cellulose with sodium caseinate/bile salt, lipid hybrid NPs, and microemulsions improved its bioavailability allowing reduction in dose, related cardiotoxicity, and crystallization (Margulis-Goshen et al., 2011; Tan et al., 2011; Morgen et al., 2012). Naturally derived phytochemicals are widely studied as potential chemopreventive agents for their pleiotropic effects and non-toxicity (Thomasset et al., 2007; Zubair et al., 2017; Wong et al., 2019). Curcumin has shown efficient chemoprotective activity in intestinal and colon cancer, but has minimal water solubility, poor absorption, and low bioavailability. To overcome this issue, curcumin-whey protein nanocapsules were developed that not only showed >70% release in 48 h but also exhibited enhanced cell internalization and bioavailability. (Jayaprakasha et al., 2016). In another study, it was revealed that curcumin encapsulated in polymeric nanocarrier improved the solubility of curcumin and showed significantly reduced number of tumors, less structural abnormalities and beta-catenin (a key intracellular messenger in gastrointestinal tract malignancies) in curcumin NPs-treated group when compared to the curcumin (Alizadeh et al., 2012).

#### Pancreatic Cancer

Pancreatic cancer is the third leading cause of all cancer-related deaths in the United States (American Cancer Society, 2019a). Late diagnosis, faster progression, low 5-year survival rate (merely 9%), and high risk of relapse make pancreatic cancer treatment and management challenging despite available first-line drug treatment involving use of gemcitabine combinations and Folfirinox^®^ (a drug cocktail of fluorouracil, leucovorin, irinotecan, and oxaliplatin) (Rahman et al., 2017; Malatesta et al., 2018; American Cancer Society, 2019a; Desai et al., 2019a; Desai et al., 2019b). Hence, chemoprevention has gained wide attention as an alternative strategy to control the occurrence and relapse.

Lipid nanocarriers have been widely investigated for this purpose, Prabhu et al. developed solid lipid NPs comprising aspirin, curcumin, and free sulforaphane as a nanocombination chemoprevention platform. The developed formulation showed significant enhancement in inhibition in Panc-1 and Mia PaCa-2 cell line models and synergism due to use of multiple drugs eliciting chemoprevention activity *via* variable mechanisms. Further, an *in vivo* chemoprevention study using *LSL-Kras* ^*G12D/*+^; *Pdx-1* ^*Cre/*+^ transgenic mice indicated significant reduction in tumor incidence with the combination nanoformulation compared to control (Sutaria et al., 2012; Thakkar et al., 2013). In another study, self micro-emulsifying drug delivery system (SMEDDS) of classical antihistaminic drug loratadine and sulforaphane was reported with enhanced oral bioavailability and chemoprevention potential in Panc-1 and Mia PaCa-2 PC cell lines (Desai et al., 2019b). Based on similar rational liposomes, dendrimers, micelles of potent chemopreventive phytochemical like curcumin, ellagic acid, etc. have been reported to show enhanced inhibition in pancreatic cancer cell lines and their application can be extended for pancreatic cancer chemoprevention (Song et al., 2011; Kesharwani et al., 2015; Wei et al., 2017).

Gene therapy has also been investigated for prevention purpose and various siRNAs, viral vectors have been studied (Lebedeva et al., 2008; Sarkar et al., 2014; Lei et al., 2017). In an interesting study by Fisher et al., replication incompetent adenoviruses capable of delivering a melanoma differentiation associated gene-7/Interleukin-24 (mda-7/IL-24) in presence of perillyl alcohol was developed. The formed nanoviral vector exhibited synergistic inhibition of pancreatic cancer cells with antitumor “bystander” response leading to suppression of primary as well as distant tumor growth. Hence, this strategy can be considered as a future clinical solution for chemoprevention and treatment and can also play a critical role in arresting pancreatic cancer relapse (Lebedeva et al., 2008; Sarkar et al., 2014).

#### Breast Cancer

Breast cancer has highest incidence and is listed to be the fourth leading cause of cancer-associated deaths in the United States (American Cancer Society, 2019a). Though chemotherapy using drugs like selective estrogen receptor modulators (tamoxifen, raloxifene, etc.), aromatase inhibitors (exemestane, anastrozole, letrozole, etc.) have shown treatment efficacy, very high incidence of breast cancer warrants development of promising preventive strategies (Ales-Martinez et al., 2015; Decensi et al., 2015; Locatelli et al., 2018). In recent years, natural products and some antineoplastic agents such as tamoxifen or raloxifene have displayed potential in chemoprevention of breast cancer (Mitra and Dash, 2018; Uramova et al., 2018). However, to enhance drug stability, achieve sustained drug release and to circumvent side effects, delivery of these agents using nanoformulations has been warranted. According to the reports, various nanoformulations such as liposome, nanofibers, nanocapsules, and NPs have been developed and investigated for prevention of breast cancer cells proliferation, breast cancer recurrence and metastasis after chemotherapy (Li et al., 2011; Roy et al., 2015; Shirode et al., 2015; Ding et al., 2016). A polymeric NPs formulation of curcumin (NanoCurc) was designed and studied to significantly attenuate incidence of mammary tumors in a rodent chemical carcinogenesis model, confirming its breast cancer chemoprevention potential in at-risk populations (Chun et al., 2012). In another study, the composite polycaprolactone/silk fibroin nanofibrous scaffolds loaded with titanocene were developed and reported to have potential for preventing the proliferation of breast cancer cells (Laiva et al., 2015). Interestingly, dietary soy isoflavones (genistein, etc.) have shown potential in reducing cancer incidence and their nanoformulations like PEGylated silica NPs, Chitosan NPs have shown significant enhancement in breast and cervical cancer inhibition (Sarkar and Li, 2003; Cai et al., 2017; Pool et al., 2018). Also, poly (ethylene glycol)-modified chitosan NPs were synthesized to encapsulate and deliver small interfering RNA (siRNA). The siRNA loaded NPs showed 4T1 cell inhibition both *in vitro* and *in vivo* ensuring its efficacy in reduction of tumor growth and metastasis (Sun et al., 2016). Wan et al. developed the lapatinib-loaded human serum albumin NPs that exhibited a core-shell structure with stealth properties preventing brain metastasis from triple-negative breast cancer (Wan et al., 2016). Interestingly, overcoming drug resistance and increasing cancer cell sensitivity towards drugs have also been investigated under this umbrella using a glycolipid-like nanocarrier encapsulating anti-tumor drug doxorubicin, which restricted drug resistance upon long-term use (Meng et al., 2019).

Hybrid NPs have also been explored for chemoprevention. Tran et al. developed the hyaluronic acid coated solid lipid NPs for co-delivery of ibuprofen and paclitaxel that resulted in synergistic inhibition on the proliferation of cancer cells (Tran et al., 2017). Zhang et al. designed a multifunctional hybrid nanomedicine integrating multiple FDA-approved modalities like radiotherapy, chemotherapy, photothermal therapy, and immunotherapy, which demonstrated elimination of the primary breast tumor and efficiently prevented tumor recurrence and metastasis to lung (Zhang et al., 2019b). Further, small peptide T4 (NLLMAAS) has been reported to inhibit tyrosine kinase, immunoglobulin, and epidermal growth factor homology-2 (Tie2), required for blood vessels reconstruction during tumor recurrence. To achieve this inhibition effectively and in targeted manner, selective NPs comprising dual-responsive amphiphilic peptide (mPEG1000-K (DEAP)-AANNLLMAAS) were developed. The NPs were capable of releasing the peptide T4 under acidic tumor environment and could achieve targeted inhibition resulting in breast tumor relapse inhibition (Zhang et al., 2019a). In another study, nanographene oxide-methylene blue formulations in combination with photodynamic and photothermal treatment were reported to prevent breast tumor regrowth and metastasis to the liver, lung, and spleen (Dos Santos et al., 2018).

#### Miscellaneous Cancers

Chemoprevention has also been studied in other less common forms of cancers including but not limited to head, neck, skin, prostate, liver. Head and neck squamous cell carcinoma is a fast progressive form of cancer and oral cancer is highly prevalent subtype therein (Crooker et al., 2018). Recently, indigenous extracellular vesicles like exosomes, microvesicles, apoptic bodies derived from mammalian or tumor cells are gaining wide attention as chemopreventive and treatment tools. They have been recognized as valuable carriers for drugs like paclitaxel, RNAs, peptides, etc. and have shown potential in inhibiting of various types of cancers (Wang et al., 2017; Han et al., 2019; Rahbarghazi et al., 2019; Wu et al., 2019)

For the site-specific local treatment and chemoprevention of oral squamous cell carcinoma, several polymeric drug delivery systems have been developed using nanotechnology which has shown enhanced activity (Desai, 2018; Ketabat et al., 2019). Some studies include drugs nanoformulations such as naringenin NPs, ellagic acid chitosan NPs, which showed significant enhancement in both bioavailability and efficacy (Arulmozhi et al., 2013; Sulfikkarali et al., 2013; Desai, 2018). In addition, cisplatin when encapsulated in polymeric micelles was reported to eliminate cisplatin induced nephrotoxicity (Endo et al., 2013; Desai, 2018). Further, PEGylated nanoliposomes of paclitaxel, resveratrol, and 5-fluorouracil were reported to show controlled drug release in inhibition of head and neck carcinoma and liposomal formulation of irinotecan (Onivyde^®^) has already been in market for pancreatic cancer management (Nie et al., 2011; Desai, 2018). Another natural chemopreventive agent, salvianolic acid B was encapsulated in phospholipid complex loaded NPs and the studies showed significant increase in intracellular uptake and improved cell inhibition when compared to drug for head and neck carcinoma (Li et al., 2016).

In the past few years, green tea and its major polyphenol, catechin have been demonstrated to superior chemoprevention activity on multiple cancer types mainly because of their antioxidant/pro-oxidant properties (Naponelli et al., 2017). To improve the drug's bioavailability, stability, and tumor selectivity, nanotechnology-based drug delivery systems have been widely studied (Tyagi et al., 2017). To study the chemoprevention efficacy of combination, gold-conjugated green tea NPs were designed that demonstrated selective toxicity towards Ehrlich's Ascites Carcinoma and breast cancer cells MCF-7 and interestingly had hepatoprotective behavior against the tumor-induced cellular damage (Mukherjee et al., 2015). For prostate cancer prevention and therapy, targeted EGCG polymeric NPs were developed using a biocompatible polymer polylactic-co-glycolic acid–polyethylene glycol-A (PLGA-PEG-A) which have a specific binding and high inhibitory action against prostate cancer cells *via* specific membrane antigen resulting in enhanced bioavailability, limited toxicity, and in turn enhanced efficacy (Sanna et al., 2017).

Hesperetin, a bio-flavonoid, plays a potential role in liver cancer management. To overcome it poor solubility, bioavailability, biocompatibility issues, hesperetin, loaded gold NPs were designed. These NPs demonstrated significantly higher *in vivo* prevention activity against lipid per-oxidation, hepatic cell damage in diethylnitrosamine-induced liver cancer model compared to the drug alone (Gokuladhas et al., 2016).

In the area of skin cancer prevention, nanotechnology-based drug formulation such as nanoemulsion of 5-fluorouracil, bromelain polymeric NPs using PLGA, solid lipid NPs of doxorubicin, 5-flurouracil have been reported (Bhatnagar et al., 2015; Shakeel et al., 2015; Ravikumar and Tatke, 2019). Use of NPs to enhance the skin deposition of chemopreventive agents is an ideal way to enhance the chemopreventive efficacy. Such examples include shell-enriched solid lipid NPs of 5-fluorouracil, curcumin-ceramide niososmes, etc. (Heenatigala Palliyage et al., 2019; Ravikumar and Tatke, 2019). NPs have also been developed and studied to elicit enhanced protection against UV radiation. Several studies including development of ultra-flexible NPs of an antioxidant diindolylmethane derivative, silver NPs, etc. (Boakye et al., 2016; Bagde et al., 2018).

### Regulatory, Clinical Insights, and Future Directions

Application of nanotechnology in cancer chemoprevention has certainly proven its potential to deliver the drugs in more effective, safer, and targeted manner. The research in this area is further advancing towards development of nanovaccines for cancer prevention. Also, early detection techniques using nanoplatforms capable of identifying pre-malignant markers are gaining attention as a preventive measure and nanodevices comprising nanochips, nanodots, quantum dots, nanoshells, and nanotubes have been reported (Bentolila et al., 2009; Boisselier and Astruc, 2009; Larocque et al., 2009; Singh et al., 2018; Facciola et al., 2019; Kheirollahpour et al., 2019; Shen et al., 2019). Despite of such advances in research, their bench-to-bedside translation for cancer prevention has a long way to go owing to regulatory and clinical considerations. In this context, mainly NSAIDS, retinoids, cyclooxygenase inhibitors, etc. have shown clinical potential through randomized clinical studies. However, more concentrated efforts and well-planned studies with measurable clinical outcomes are warranted. Further, proving the safety of nanoformulations is an urgently needed aspect. In view of regulatory approval of nanotechnology-based products for cancer treatment and other conditions, we should expect the clinical translation of nanotechnology-based products for cancer chemoprevention in near future.

## Author Contributions

PD, JW, and SP conceived and proposed the idea. PD compiled the manuscript with support from NT, PW, SZ. DA, JW and SP reviewed and revised the manuscript.

## Conflict of Interest

The authors declare that the research was conducted in the absence of any commercial or financial relationships that could be construed as a potential conflict of interest.
